# Cystic Fibrosis Patients Infected With Epidemic *Pseudomonas aeruginosa* Strains Have Unique Microbial Communities

**DOI:** 10.3389/fcimb.2020.00173

**Published:** 2020-04-24

**Authors:** Nicole Acosta, Barbara Waddell, Alya Heirali, Ranjani Somayaji, Michael G. Surette, Matthew L. Workentine, Harvey R. Rabin, Michael D. Parkins

**Affiliations:** ^1^Department of Microbiology, Immunology and Infectious Diseases, University of Calgary, Calgary, AB, Canada; ^2^Department of Medicine, University of Calgary, Calgary, AB, Canada; ^3^Departments of Biochemistry and Biomedical Sciences, McMaster University, Hamilton, ON, Canada; ^4^Faculty of Veterinary Medicine, University of Calgary, Calgary, AB, Canada

**Keywords:** cystic fibrosis, microbiome, *Pseudomonas aeruginosa*, PES, epidemic, infection

## Abstract

*Pseudomonas aeruginosa* is the archetypal cystic fibrosis (CF) pathogen. However, the clinical course experienced by infected individuals varies markedly. Understanding these differences is imperative if further improvements in outcomes are to be achieved. Multiple studies have found that patients infected with epidemic *P. aeruginosa* (ePA) strains may have a worse clinical prognosis than those infected with unique, non-clonal strains. Additionally, the traditionally uncultured CF lung bacterial community (i.e., CF microbiome) may further influence the outcome. We sought to identify if these two important variables, not identified through routine culture, associate and together may contribute to disease pathogenesis. Patients were classified as being infected with Prairie Epidemic ePA (PES) or a non-clonal strain, unique PA strains (uPA), through a retrospective assessment of a comprehensive strain biobank using a combination of PFGE and PES-specific PCR. Patients were matched to age, sex, time-period controls and sputum samples from equivalent time periods were identified from a sputum biobank. Bacterial 16S rRNA gene profiling and *Pseudomonas* qPCR was used to characterize the respiratory microbiome. We identified 31 patients infected with PES and matched them with uPA controls. Patients infected with PES at baseline have lower microbial diversity (P = 0.02) and higher *P. aeruginosa* relative abundance (*P* < 0.005). Microbial community structure was found to cluster by PA strain type, although it was not the main determinant of community structure as additional factors were also found to be drivers of CF community structure. Communities from PES infected individuals were enriched with *Pseudomonas, Streptococcus* and *Prevotella* OTUs. The disproportionate disease experienced by ePA infected CF patients may be mediated through a combination of pathogen-pathogen factors as opposed to strictly enhanced virulence of infecting *P. aeruginosa* strains.

## Introduction

Cystic fibrosis (CF) lung disease is characterized by thickened secretions that impair mucociliary function, thereby reducing the clearance of inhaled material (Gibson et al., 2003). The CF lung is particularly susceptible to infections by *Pseudomonas aeruginosa*, a common CF pathogen, which historically infected 60–80% of CF patients (Lambiase et al., 2006; Razvi et al., 2009; Crull et al., 2018) and this accounts for most CF-associated morbidity and mortality (Emerson et al., 2002; Schluchter et al., 2002; Gibson et al., 2003; MacKenzie et al., 2014). However, due to the use of early eradication treatments there is now a declining prevalence of *P. aeruginosa* infection which has been correlated with the increased prevalence of *Staphylococcus aureus* (Cystic Fibrosis Foundation Patient Registry: 2018 Annual Data Report, 2019). *P. aeruginosa* virulence and its conversion to a hyper-alginate producing mucoid phenotype during chronic infections is further associated with progressive decline in lung function, increased risk of hospitalization and reduced survival (Henry et al., 1992; Emerson et al., 2002; Li et al., 2005).

The last two decades have distinguished that even amongst patients with chronic *P. aeruginosa* infection that marked heterogeneity in clinical outcomes occur. The use of molecular methods have established that clusters of patients infected with clonal *P. aeruginosa* strains exist within geographically defined patient populations and particularly prevalent strains associated with patient-patient transmission have been termed epidemic (ePA) (Parkins et al., 2018). Some notable examples include the Liverpool Epidemic Strain (LES)—prevalent in the United Kingdom (UK) and Eastern Canada, the Midlands-1 strain (identified in the UK), Houston-1 (identified in United States), and several Australian Epidemic Strains (AUST 01-04) (Cheng et al., 1996; Armstrong et al., 2002; Al-Aloul et al., 2004; O'Carroll et al., 2004; Scott and Pitt, 2004; Luna et al., 2013; Middleton et al., 2018). Recently, our research group identified a novel strain of *P. aeruginosa*, termed the Prairie Epidemic Strain (PES), infecting CF patients attending the Calgary Adult CF Clinic (CACFC) (Parkins et al., 2014). PES was found to represent ~30% of chronically *P. aeruginosa* infected CF adult patients within the clinic and was identified infecting patients newly transitioning from neighboring Prairie-based Canadian CF centers over 25 years, suggesting broad endemicity (Somayaji et al., 2017; Middleton et al., 2018).

PES strains have increased antimicrobial resistance compared to unique strains (Parkins et al., 2014). Patients infected with PES experience disproportionate disease progression and infection with PES has been associated with an increased risk of respiratory related death and/or lung transplantation (Pritchard et al., 2016; Somayaji et al., 2017). However, a comparative study that screened a large panel of ePA isolates (including PES) against unique strains from CF patients for virulence traits including virulence factor production, biofilm formation, planktonic growth, mucoidy, and antibiotic susceptibility, showed that PES isolates do not behave significantly differently than unique/non-clonal isolates, albeit these were conducted *in vitro* and may not be reflective of the complexity of the lung environment (Duong et al., 2015). Thus, far the mechanism by which ePA associates with a worsened prognosis remains unknown.

More recently, it has been demonstrated that the lower airways of CF patients are infected with a highly diverse and complex microbial community constituting personalized mini-ecosystem (Surette, 2014). It has been shown that microbial diversity correlates with disease status, genotype and sex in CF patients, where the burden of antibiotic treatments appears to be a major driver of this (Cox et al., 2010; Klepac-Ceraj et al., 2010; Fodor et al., 2012; Zhao et al., 2012, 2014; Carmody et al., 2013; Renwick et al., 2014; Boutin et al., 2015; Coburn et al., 2015). Recent studies have even shown that microbiome-based biomarkers are better predictors of long-term clinical outcomes (Acosta et al., 2018) and treatment response (Heirali et al., 2017, 2019) than traditional bacterial culture. The role of individual constituent pathogens in the CF microbiome and the progression of CF lung disease is an area of significant research interest. To date, however, studies assessing how these two important modifiers of CF lung disease, ePA and the CF microbiome, intersect and influence one another have yet to be conducted. Herein we present a comparative case-control study of the microbiome of CF patients infected with a particular ePA, PES, to determine if their microbiota differed from patients infected with unique strains. We hypothesized that the clinical disadvantage experienced by PES infected patients may in part be attributed to fundamental differences in the bacterial composition of their lower airways microbiota.

## Materials and Methods

### Ethics Statement

This study was approved by the University of Calgary Conjoint Regional Health Ethics Board (REB15-2744 and REB15-0854). For all samples within the Calgary Adult CF (CACFC) sputum and strain biobanks, informed consent was provided by each and every patient prospectively for all samples collected from 2005-present. Samples collected before this date (including those from patients who subsequently consented) were materials directed for clinical care—and remaining samples used for microbiota analysis—and thusly individual consent was waived by local ethics board. All consents were informed, with extensive discussions with each patient. In all cases consent was documented with in a written format using an institutionally approved document. All samples were de-identified, and their data presented in aggregate.

### Patients and Sample Selection

CACFC maintains two separate biobanks; a longitudinal sputum biobank containing whole sputum samples from patients collected since 1998 and a comprehensive strain biobank including every cultured pathogen identified dating back to 1978. We combined data from these two collections to determine if the microbiome of patients infected with PES, a specific ePA strain, differed from those infected with non-clonal strains (here we excluded patients infected with known ePA strains other than PES). Infecting strain type was determined during previous work from our group using frozen strains recovered from the comprehensive strain biobank with complementary strain typing techniques including pulse-field gel electrophoresis (PFGE) and multi-locus sequence typing (Parkins et al., 2014). For inclusion in the study, patients had to have met the Leeds criteria for chronic *P. aeruginosa* infection (Lee et al., 2003). Patients identified to be infected with the ePA (i.e., PES) were identified and matched by birth period (+/− 1 year) and sex to control patients infected with unique non-epidemic strains of PA (uPA). We then examined our longitudinal sputum biobank to identify sputum samples collected at similar time periods (±3 months) between the two patient groups for up to three time points for the analysis. These time points included T1 (the earliest available sample within the biobank that met the criteria mentioned above), T3 (the latest available sample), and T2 (intermediate sample). Samples were excluded if they were collected within 2 weeks of systemic antibiotics or PEx due to the confounding effects of antimicrobials on communities. PEx events were identified based on clinical chart review and PEx was defined as previously described by Lam et al. (2015). To confirm the previous classification with respect to ePA vs. uPA (Parkins et al., 2014) in our sample population, a previously described PCR assay (Workentine et al., 2016) targeting a specific region located on novel 40-kb genomic island on the PES strains was amplified from the total DNA isolated from frozen whole sputum (see below). Patient's characteristics including demographics, baseline lung function, CF comorbidities and cultured pathogens were captured. Based on the percent predicted forced expiratory volume in one second (FEV_1_%) at sample collection patients were categorized in the following stages of lung disease: mild (>80%), moderate (40–80%), and advanced (<40%).

### DNA Extraction, Amplification, and Sequencing

Total genomic DNA was extracted as previously described by Acosta et al. (2017) from 144 sputum samples. Blank samples containing only ultra-pure distilled water (Invitrogen; Thermo Fisher Scientific, Inc.) were used as control for contamination in the DNA extraction, amplification and sequencing steps. All samples and controls were amplified by PCR in triplicate using barcoded primer pairs flanking the V3-V4 region of the 16S rRNA gene as previously described (Acosta et al., 2018). Pooled PCR amplicons were sequenced on the MiSeq platform (Illumina, Inc.,San Diego, CA, USA) as described previously (Acosta et al., 2017). Sequences were processed using a custom Snakemake pipeline (Timsit et al., 2016). After sequence processing a total of 14,877,996 reads (mean, 103,319 reads/sample; IQR, 87,631- 119,739) with 254 total OTUs (mean 62 OTUs/sample; range, 44–75) were generated.

### Microbial Community Composition and Diversity Analysis

All statistical analysis described below were conducted in R 3.4.3 (R Core Team, 2014). To compare bacterial community structure across both ePA and uPA groups of patients, beta diversity measures (i.e., Bray-Curtis dissimilarities and unweighted UniFrac distances) and permutational multivariate analysis of variance (PERMANOVA) statistical analysis (Anderson, 2001) were performed using phyloseq (McMurdie and Holmes, 2013) and vegan (Oksanen et al., 2017) R packages. To correct for the effect of having more than one sample per patient in our cohort, the patient variable was blocked for the PERMANOVA analysis. To visualize patterns of bacterial community structure, we performed a non-metric multidimensional scaling (NMDS) ordination of Bray-Curtis dissimilarities among the samples. Beta diversity (i.e., Bray-Curtis) was calculated after normalizing all samples proportionally (Calle, 2019). We performed two secondary analyses to account for higher rates of culture positivity in our control uPA population. In the first assessment we removed all the OTUs corresponding to the genus *Pseudomonas* and assessed the remaining community—termed here—the accessory microbiome. In the second analysis we included only those sputum samples from pairs of ePA and uPA matched patients that were both *P. aeruginosa* culture positive. Each sample from the three data sets (i.e., full data set, accessory microbiome and data set from only PA positive in culture) were also rarefied to 15,000 sequences and analyzed. We also tested for differentially abundant OTUs between ePA and uPA samples using DESeq2 (Love et al., 2014). *P*-values were adjusted for multiple comparisons by using Benjamini–Hochberg *P*-value correction for multiple testing. Log_2_ fold change of OTUs plots were created using GraphPad Prism version 7.0 (La Jolla, CA). Alpha diversity (i.e., Shannon diversity index) measure was calculated using phyloseq (McMurdie and Holmes, 2013). Dominant taxon was considered to the most abundant OTU found in each sample. Wilcoxon rank-sum (Mann-Whitney) test was used for continuous variables and the Fisher exact probability test for categorical variables.

### Quantitative PCR

Quantitative PCR (qPCR) was performed to determine the absolute abundance of *P. aeruginosa* by using the absolute quantification method. Primers used for the amplifications are described by Qin et al. (2003). The concentration of the total genomic DNA isolated from the sputum samples was measured and normalized to a concentration of 10 ng/μL. All qPCR reactions were carried out in the StepOne Plus Real-Time PCR System (Applied Biosystems). Each 10 μl reaction contained 5 μl of SsoAdvanced Universal Inhibitor-Tolerant SYBR Green Supermix (Bio-Rad), 1 μl of forward and reverse primer mix (final concentration, 250 nM), and 4 μl of the template DNA. The qPCR program reaction consisted of an initial denaturation step at 95 °C for 10 min followed by 40 cycles of 95 °C for 15 s, 68 °C for 1 min and ended with a melting curve analysis of 1 cycle of 95°C for 15 s, 60°C for 1 min, 95°C for 15 s, and 60°C for 15 s.

The absolute quantity was estimated against serial dilutions of DNA standards from *P. aeruginosa* PA01 strain that were amplified to generate standards curves. Standard curves were generated by using 1:5 dilutions of a 1 ng/μL stock of purified target template DNA. DNA extraction of *Stenotrophomonas maltophilia* was also included as no template control (NTC). For bacterial DNA extraction, 400 μl of culture was pelleted by centrifugation, followed by resuspension in 800 μl of 200 mM NaPO_4_ and 100 μl of guanidine thiocyanate-ethylenediaminetetraacetic acid-sarkosyl. The solution was then mechanically lysed by bead beating and DNA was extracted following the protocol for sputum samples as previously described (Acosta et al., 2017).

## Results

### Selection and Characteristics of ePA-uPA Match

A total of 31 patients with PES with available sputum samples met inclusion and exclusion criteria (16 male; 15 female) and matching uPA controls were identified. The control cohort consisted of 31 uPA infected patients (13 male; 18 female) and specifically excluded patients infected with any other epidemic strain. Ten patients were matched twice owing to limitations in the availability of ePA and matched controls, but the samples used in each match corresponded to different dates (Supplementary Figure 1 and Supplementary Table 1). In total we identified 36 pairs of ePA-uPA matched patients (Supplementary Figure 1). Patients were matched by birth cohort ±1 year apart (Median: 0.4, IQR: 0.2–0.7 years), by sex (19 female pairs and 17 male pairs), and by the time of sample collection (±3 months). Samples were collected a median 1.9 months (IQR 0.6–3 months) apart between patients and controls.

Clinical characteristics and demographics were analyzed at baseline (i.e., T1). Demographics at T1 of matched patients (ePA vs, uPA) were similar with respect to demographics with one exception, patients with ePA infection had disproportionally advanced airways disease (Table 1). Of note, however, this difference diminished over time as patients progressed to end-stage lung disease and were excluded, having undergone transplantation (data not shown). At T1, ePA patients were more likely to have sputum cultures yielding *P. aeruginosa*, and mucoid *P. aeruginosa* despite all patients meeting Leeds criteria for chronic infection. Chronic treatments (i.e., Azithromycin, inhaled colistin, inhaled DNase and inhaled tobramycin) did not differ between groups (data not shown). A sensitivity analysis was done to control for those cases or control patients that were matched twice in the study, in which only one match was included (Supplementary Table 1). We observed similar trends and results with respect to the clinical characteristics and demographics analyzed by time points, with the exception that there was not difference between ePA vs, uPA for the FEV_1_% predicted at sample collection (data not shown).

**Table 1:** Baseline characteristics of ePA and uPA patients.

**Variable**	**ePA (*n* = 36)**	**uPA (*n* = 36)**	***P*-value**
Demographics*		
Sex (M:F)	17:19	17:19	1
Age (years)	23.2 (19.6–28.4)	23.2 (19.1–28.9)	0.901
ΔF508 / ΔF508	19 (52.7)	20 (55.5)	1
FEV_1_% predicted	52.5 (39.5–72)	78 (55.5–85)	0.013
FVC % predicted	84.5 (67.9–99)	91.5 (75.5–106.5)	0.239
BMI (kg/m^2^)	21.2 (19.8–22.4)	20.7 (19.6–22.1)	0.524
CF related diseases*		
Pancreatic insufficiency	34 (94.4)	34 (94.4)	1
CFRD	2 (5.5)	4 (11.1)	0.674
CF-LD	6 (16.6)	6 (16.6)	1
Osteopenia/Osteoporosis	4 (11.1)	6 (16.6)	0.735
**Cultured pathogen**^**†**^
*P. aeruginosa*	36 (100)	27 (75)	0.002
Mucoid *P. aeruginosa*^‡^	36 (100)	20 (74.07)	0.002
*Bcc*	0 (0)	2 (5.5)	0.493
*S. aureus*	10 (27.7)	18 (50)	0.09
*H. influenzae*	1 (2.7)	4 (11.1)	0.357

* *Data are presented as n (%) or median (inter-quartile range)*.

*Data are presented as n (%) of samples that were positive in culture*.

‡ *The proportion was calculated out of patients with P. aeruginosa positive culture, therefore data are presented as n (%) of patients with mucoid PA [ePA (n = 36) and uPA (n = 27)]*.

*Fisher exact probability test at a two-tailed or Wilcoxon rank-sum (Mann-Whitney) tests were performed*.

*ePA, Epidemic strains of PA (ePA); uPA, unique PA; BMI, body mass index; CFRD, Cystic fibrosis (CF)-related diabetes; CF-LD, CF liver disease; Bcc, Burkholderia cepacia complex. Both CFRD and CF-LD were defined in real time at each clinic visit by the attending clinician*.

### Microbial Communities Are PA Strain Type Specific

**Figure 1:**
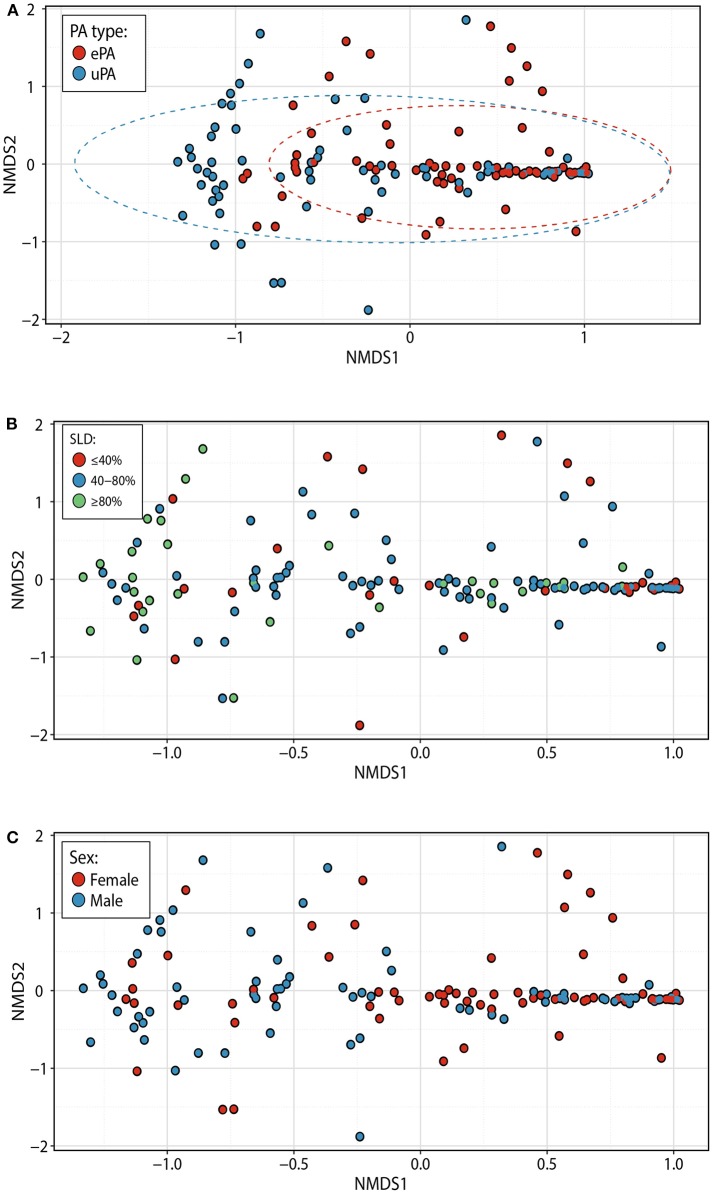
Bacterial community structure within patients with epidemic and unique *P. aeruginosa* strains. NMDS plots based on Bray-Curtis distances among 144 samples (representing each point). Samples are colored based on PA typing [ePA (red) and uPA (blue)] **(A)**, the stage of lung disease at the sample collection [<40% (advanced), 40–80% (moderate), and >80% (mild)] **(B)**, and gender [female (red) and male (blue)] **(C)**. Ellipses denote 95% confidence intervals. NMDS, non-metric multidimensional scaling, PA, *P. aeruginosa*; ePA, Epidemic strains of PA; uPA, unique strain of PA; SLD, stage of lung disease.

To confirm *P. aeruginosa* strain classification, each sputum subjected to microbiome analysis underwent PES-specific PCR. For each patient identified as ePA the PES PCR was positive, and for patients identified as uPA they were all negative, confirming the previous classification of all patients (Supplementary Figure 2 and data not shown). To understand any potential differences in the CF microbiome of patients with ePA and uPA, we compared microbial communities by calculating the Bray-Curtis dissimilarity after proportionally normalizing all samples. NMDS plots of all 144 sputum samples illustrate the clustering of samples by the PA typing (Figure 1A), the stage of lung disease [mild (>80%), moderate (40–80%), and advanced lung disease: (<40%)] (Figure 1B), and by sex (Figure 1C). To test these, PERMANOVA statistical analysis was performed and showed that the composition of the microbial communities was found to be distinguishable by PA infecting strain type (*P* = 0.002), FEV_1_% (*P* = 0.002), and sex (*P* = 0.002). Although their relative influence on microbial composition was smaller (i.e., PA infecting strain type (*R*^2^ = 4.6%), FEV_1_% (*R*^2^ =1.6%), and sex (*R*^2^ = 2.6%)], we sought to determine whether there was an interaction between the PA infecting strain type and FEV_1_% at sample collection. There was not a significant difference in the microbial composition based on the interaction between both variables (*F* = 2.23, *R*^2^ = 1.4%, *P* = 0.062). However, when samples were categorized by their stage of lung disease, we observed that there was an interaction between PA infecting strain type and their disease-stage (*F* = 2.47, *R*^2^ = 3.2%, *P* = 0.021). Similar results were found when the data set was rarefied (Supplementary Table 2) or when a sensitivity analysis was done to control for those cases or control patients that were matched twice in the study (data not shown). Consistent with most published data, we found that the strongest driver of CF community structure was by patient (*F* = 4.2, *R*^2^ = 56.3%, *P* = 0.002). To further test the effect of patient in the above analysis, we first blocked the metadata by the patient factor and then we used PERMANOVA test to determine the effect of PA infecting strain type, FEV_1_%, and sex on the microbial communities. Congruent with our previously stated findings, after blocking by patient factor PA infecting strain type, FEV_1_%, and sex contributed significantly to community composition (*P* = 0.019 for all three factors). For each of the 36 pairs of ePA-uPA matched patients we identified up to three time points [i.e., early (T1), intermediate (T2) and late (T3)], to further test whether the length of time followed (Supplementary Figure 1) may have an impact in the composition of the microbial communities. PERMANOVA statistical analysis was performed and showed that the microbial communities were not found to be distinguishable by the length of infection (*F* = 1.59, R^2^ = 1.1%, *P* = 0.136). Similar results were found when data was stratified by either Patient ID (*F* = 1.59, *R*^2^ = 1.1%, *P* = 0.298) or when the rarefied data (*F* = 1.50, *R*^2^ = 1.06%, *P* = 0.193) was analyzed. Finally, there was not a significant difference in the microbial composition based on the interaction between sampling time-period and PA infecting strain type (*F* = 0.25, *R*^2^ = 0.17%, *P* = 0.951).

To further understand effects of lung function status on the CF microbiome of patients with ePA and uPA infection, we identified the pairs of ePA-uPA that at anytime point (i.e., T1, T2, and T3) had a similar stage of lung disease (i.e., mild, moderate, and advanced). Out of the initial 36 pairs of ePA-uPA, we found the following number of pairs with a similar stage of lung disease: mild (*n* = 4 pairs), moderate (*n* = 17 pairs), and advanced (*n* = 2 pairs). We then compared their microbial communities by calculating the Bray-Curtis dissimilarity when it was controlled for lung disease stage. PERMANOVA statistical analysis of this smaller dataset showed that the composition of the microbial communities was found to be distinguishable by PA infecting strain type only in the mild group for all time points (*F* = 5.6, *R*^2^ = 41.2%, *P* = 0.015) and in the mild group at T1 time point (*F* = 6.07, *R*^2^ = 50.3%, *P* = 0.026) (similar results when data was rarefied, data not shown) but not for the moderate (*F* = 0.7, *R*^2^ = 1.76%, *P* = 0.552). For the advanced group, we did not have an appropriate number of samples to do the PERMANOVA test.

**Figure 2:**
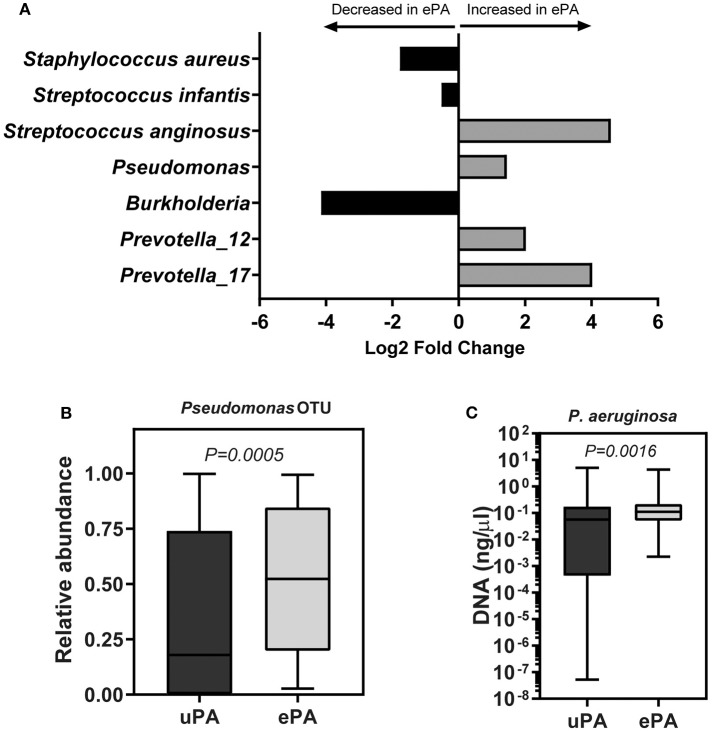
OTUs that were significantly different between samples with epidemic and unique *P. aeruginosa*. Log_2_ fold change of OTUs that were significantly abundant between ePA and uPA samples calculated by using DESeq2 (*P* < 0.05, Wald test, and false discovery rate). Analysis was done using only the top 20 OTUs which accounted for >0.2 % of total abundance for the whole data **(A)**. Relative abundance of *Pseudomonas* OTU reads between ePA and uPA samples, Wilcoxon rank-sum (Mann-Whitney) tests were performed **(B)**. qPCR of *P. aeruginosa* (uPA samples, 68; ePA, 71) in sputum samples of CF patients. Samples which their qPCR results were undetermined; they were not included in the illustration (graph is based on a logarithmic scale) or in the statistical analysis (Wilcoxon rank-sum (Mann-Whitney) test) **(C)**. For **(B,C)** graphs, the median and interquartile ranges (IQR) are indicated as the middle, top and bottom lines of each box. Ends of the whiskers mark the min and max. ePA, epidemic strains of *P. aeruginosa*; uPA, unique strains of *P. aeruginosa;* OTU, operational taxonomic unit.

Overall, these results indicate that the PA strain type (i.e., ePA and uPA) contributes to the clustering of the samples. To identify differentially abundant OTUs between ePA and uPA samples, a Wald test was used to determine the changes in the abundance of OTUs in the case and control cohorts. We found 7 OTUs among the top 20 OTUs (i.e., top 20 OTUs accounting for > 0.2 % of total abundance for the whole data) that were significantly different in abundance in ePA and uPA sample. *Pseudomonas, Streptococcus anginosus* and two OTUs for *Prevotella* genera were enriched in ePA samples, whereas *Streptococcus infantis, S. aureus* and *Burkholderia* were enriched in uPA (*P* < 0.05) (Figure 2A).

### Patients Infected With ePA at Baseline Have Lower Microbial Diversity

**Figure 3:**
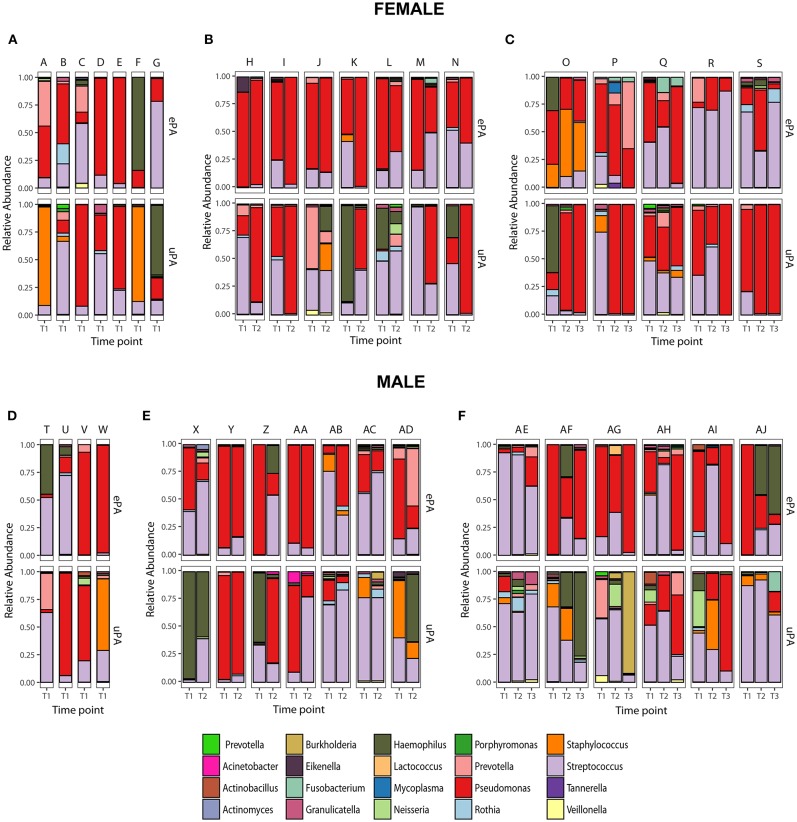
Paired Microbiota from CF patients infected with epidemic (cases) and unique *P. aeruginosa* (controls) strains. Relative abundance at the genus level of 36 match set of samples (match ID denoted as letters) from CF patients (Female: upper panel and Male: lower panel) that were analyzed at only one time-point (T1) **(A,D)**, two time-points (T2) **(B,E)** and three time-points (T3) **(C,F)**. Top 20 genera accounting for >0.2% of total abundance for the whole data set are colored and presented in the figure.

**Figure 4:**
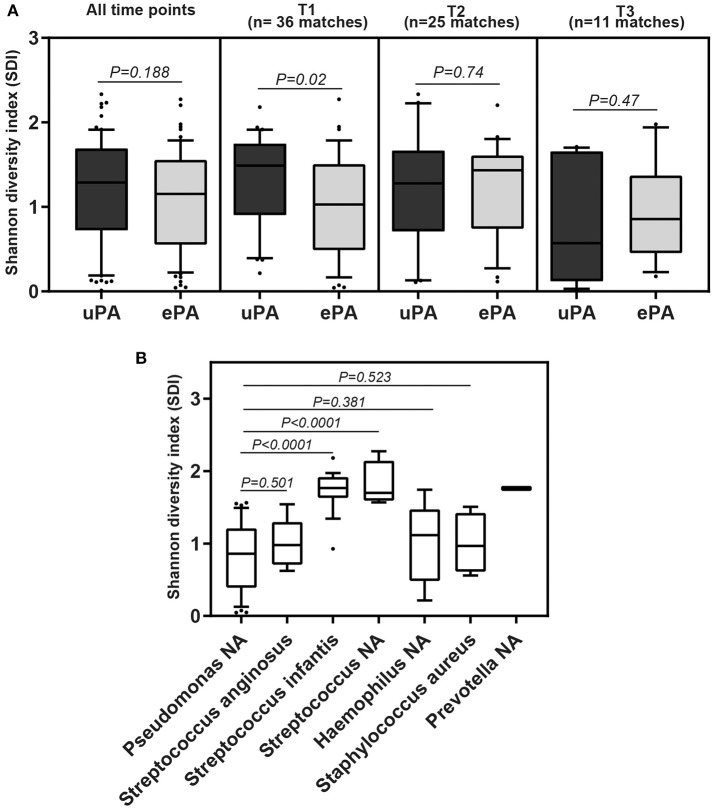
Alpha diversity from sputum samples from CF patients. Boxplot comparison of the alpha-diversity by PA strain type: epidemic (ePA) and no-epidemic *P. aeruginosa* (uPA) at all-time points, time 1, time 2 and time 3 point **(A)**, and by the most abundant OTUs found in the microbial communities at time 1 **(B)**. For those that have NA means that based on the sequencing results cannot be assigned to the species level. The median and interquartile ranges (IQR) are indicated as the middle, top and bottom lines of each box. Ends of the whiskers mark the 10 and 90 percentile in each box. Wilcoxon rank-sum (Mann-Whitney) tests were performed.

The overall microbial composition of the cohort (cases and controls), based on the total number of reads, was dominated by *Pseudomonas* (42.6%), followed by *Streptococcus* (35%), *Haemophilus* (7.8%), *Staphylococcus* (4%), *Prevotella* (3.5%), *Rothia* (1.2%), *Granulicatella* (0.8%), *Neisseria* (0.8%), *Burkholderia* (0.5%), *Veillonella* (0.4%), *Fusobacterium* (0.2%), and *Actinobacillus* (0.2%) (Figure 3). We determined changes in the evenness and richness in the CF microbiome by measuring SDI in the ePA and uPA groups. There was not statistical difference in SDI between the ePA and uPA samples (Figure 4A). However, when pooled analysis was done separately by time point, it showed that at T1 but no other times points, patients with ePA had lower SDI (Figure 4A, second panel). To further analyze the effect of the most abundant OTUs in all microbial communities on alpha diversity at baseline (i.e., T1), we compared the SDI values based on the most abundant OTUs per sample. Overall, alpha diversity was significantly lower if the samples were categorized for having *Pseudomonas* as the most abundant OTU, while samples dominated by some *Streptococcus* genera (expect for S. *anginosus*) showed the greatest diversity (Figure 4B).

### ePA Patients Have Greater Proportional Abundance of *Pseudomonas*

We found that the ePA group showed a significant increase in relative abundance of *Pseudomonas* as compared to the uPA control group (54.3 vs. 19.18%, *P* = 0.0005) (Figure 2B). To confirm the observed difference of *Pseudomonas* relative abundance between ePA and uPA samples, we quantified the amount of *P. aeruginosa* present in the samples using qPCR. In accord with our sequencing data, qPCR confirmed significantly higher abundances of *P. aeruginosa* in the ePA samples compared to uPA control samples (Figure 2C). Additionally, we found samples from ePA patients were more likely to have *Pseudomonas* as the most abundant genera (ePA: 61.1 vs. uPA: 38.9%, *P* = 0.012). We also compared the relative abundance of *Pseudomonas* between ePA and uPA patients when it was controlled for lung function as described above. Similar to the results found on beta diversity, when the effects of lung function status were controlled, here we found that in the samples of ePA-uPA patients with mild but not in moderate stage of lung disease, ePA showed an increase in relative abundance of *Pseudomonas* compared to the uPA control group (Supplementary Figure 3). Collectively, these results suggest that within CF sputum, ePA are more likely to be present in greater relative abundance than uPA.

### Accessory Microbiome Is PA Strain Type Specific

To address concerns that *P. aeruginosa* dominates microbial community analysis, we analyzed these same sputum samples after removing entirely from the dataset all the OTUs corresponding to the genus *Pseudomonas* in order to better examine the accessory microbiome. We found that samples still clustered together based on their PA strain type, FEV_1_% and patient sample. We also observed an association between PA strain type and FEV_1_% (Table 2). Similar results were found when the accessory microbiome data set was rarefied (Supplementary Table 3).

**Table 2:** CF bacterial community structure variation explained by various factors and their interactions based on Bray-Curtis distances using the accessory microbiome data set.

**Variable**		***F***	***R*^**2**^ (%)**	*P*-value*
Single Factor	A) PA strain type	3.29	1.24	0.003
	B) FEV_1_%	2.3	0.87	0.047
	C) Sex	2.01	0.76	0.059
	D) Stage of lung disease^†^	1.23	1.7	0.208
	E) Patient	2.7	60.53	0.003
Interaction	A x B	2.64	1.8	0.020
	A x D	1.7	2.34	0.047

* *P-values determined by PERMANOVA which were adjusted for multiple testing by using the procedure of Benjamini and Hochberg (false discovery rate threshold, 5%)*.

*An “x” represents interaction between variables that are letter-coded*.

*Stages of lung disease: mild (>80%), moderate (40–80%), and advanced (<40%)*.

*PA, P. aeruginosa*.

Furthermore, we used a complementary approach to compensate for the domination of communities by *Pseudomonas* OTU. Our previous analyses were performed using Bray-Curtis distance metrics, which takes into account both community membership and relative abundance. Here we used unweighted Unifrac metrics, which emphasizes rare organisms as it only considers presence or absence of OTUs and not relative abundance, to determine factors that may influence the variation in CF phylogenetic community structure. PA strain type showed a similar trend when explaining the variation in microbial community structure (*F* =1.8, *R*^2^ =1.25%, *P* = 0.049; PERMANOVA).

Finally, because there were differences in *P. aeruginosa* real-time growth and baseline mucoidy between the ePA and uPA groups at the time of sample collection, we performed subgroup analysis where we excluded those who were *P. aeruginosa* culture negative (Table 1). After subgrouping, we identified 33 pairs of matched ePA-uPA patients corresponding to 116 sputum samples (58 ePA and 58 uPA). Similar to our whole data set, we found that microbial community composition still clustered by PA strain-type, FEV_1_%, sex, and patient sample (Table 3). Similar results were found when the data set was rarefied (Supplementary Table 4). To analyze the effect of mucoid phenotype on microbial composition we performed PERMANOVA statistical analysis. Although its relative influence was smaller, mucoid phenotype (*R*^2^ = 2.1%) was also a significant driver of CF bacterial community structure (*P* = 0.036).

**Table 3:** CF bacterial community structure variation explained by various factors and their interactions based on Bray-Curtis distances in 116 samples that were *P. aeruginosa-*culture positive.

**Variable**		***F***	***R*^**2**^ (%)**	*P*-value*
Single Factor	A) PA strain type	8.61	2.27	0.002
	B) FEV_1_%	9.74	2.57	0.002
	C) Sex	8.15	2.15	0.002
	D) Stage of lung disease^†^	2.53	4.29	0.033
	E) Patient	4.51	63.1	0.002
	F) Mucoid PA	2.49	2.14	0.040
Interaction	A x B	2	1.65	0.081
	A x D	2.27	3.7	0.033

* *P-values determined by PERMANOVA which were adjusted for multiple testing by using the procedure of Benjamini and Hochberg (false discovery rate threshold, 5%)*.

*An “x” represents interaction between variables that are letter-coded*.

*Stages of lung disease: mild (>80%), moderate (40–80%), and advanced (<40 %)*.

*PA, P. aeruginosa*.

## Discussion

Several ePA infecting individuals with CF have been identified and many have been associated with worse patient outcomes. The PES strain, in particular has only recently been described, but is now known to have existed within CF populations since at least 1979 (Parkins et al., 2014; Somayaji et al., 2017). In a longitudinal study of CF adults, patients with chronic PES infection had a 4.16 greater hazard ratio for progression to death/lung transplantation compared to those without *P. aeruginosa*, and 1.77 greater hazard ratio to those infected with unique *P. aeruginosa* strains (Somayaji et al., 2017). Why ePA are associated with adverse outcomes is unknown. Several comparative studies amongst ePA have been conducted. No individual phenotype or genetic marker has been consistently associated with ePA status (Jeukens et al., 2014; van Mansfeld et al., 2016). Indeed, no particular phenotype was found to exist disproportionally in PES and other epidemic strains relative to unique strains of *P. aeruginosa* (Duong et al., 2015). A wide range of phenotypic heterogeneity has been observed amongst PES, even in isolates from a single patient (Workentine et al., 2013), as is typical for chronically infecting CF *P. aeruginosa* populations, thus making comparisons between individual isolates difficult to interpret. Herein, we hypothesized that through the modification of the lower airways microbial community structure, PES may select for a community that is more harmful to the host. Indeed, we suspect that this may be a primary means of ePA pathogenesis. These may occur through the production of a number of secondary metabolites that act to shape the surrounding community structure (Tashiro et al., 2013; Beaume et al., 2015; Stubbendieck and Straight, 2016). This aligns with our main observation that PES is more likely to dominate CF respiratory communities than uPA strains, suggesting that PES may have an enhanced competitive advantage in airway communities. This is in keeping with the ability of PES and epidemic strains to “displace” previous infecting uPA during super-infection events (Fothergill et al., 2012; Parkins et al., 2014, 2018; Oluyombo et al., 2019).

We observed strain status to be an important determinant of community structure. In particular, PES microbial communities were enriched with organisms such *Pseudomonas, S. anginosus*, a member of the *Streptococcus anginosus*/milleri group (SMG) and two OTUs corresponding to the *Prevotella* genus. SMG members may play a role in the progression of the CF lung disease by either direct pathogenesis or upregulating the virulence factors of classic pathogens, such as *P. aeruginosa* (Sibley et al., 2008; Hill, 2017). The genus *Prevotella* has been shown to be a core member of the CF microbiome (Surette, 2014). In addition to presumptive roles in direct pathogenesis and induction of an exaggerated immune response (Ulrich et al., 2010), *Prevotella* spp. may alter the airways environment through the production of antibiotic modifying enzymes and protect *P. aeruginosa* from exogenous insults (Sherrard et al., 2016; Gilpin et al., 2017). On the other hand, we found that uPA communities were enriched by *S. aureus, S. infantis* and *Burkholderia* OTU. *S. aureus* is often one of the first microorganism detected in infants and children with CF (Nixon et al., 2002). As CF patient's transition from childhood into adulthood, the prevalence of *S. aureus* decreases but it remains significant during adulthood, displaying a negative correlation with the prevalence *P. aeruginosa* (Cystic Fibrosis Foundation Patient Registry: 2016 Annual Data Report, 2017). Indeed, the prevalence of PES has previously been shown to negatively associate with MSSA infection (Somayaji et al., 2015), which may suggest that PES negatively selects against *S. aureus* through direct or indirect means. Multiple other studies have shown the potential for both positive and negative interactions between *P. aeruginosa* and *B. cepacia* complex (BCC). For example, members of the BCC can form biofilms with *P. aeruginosa* that allow network of interactions (Eberl and Tümmler, 2004). On the other hand, it has been also shown that *P. aeruginosa* can inhibit growth of *B. cenocepacia* (Bragonzi et al., 2012; Costello et al., 2014). Whether this interaction may be strain specific is, as yet, unknown. However, rates of *B. cenocepacia* infections among CACFC patients have historically been lower than those among neighboring clinics suggesting that PES could be antagonistic.

Importantly, PA strain type was not the main determinant of CF bacterial community structure; this driver only explained ~5% of the observed variation. In fact, the major determinant, explaining more than 50% of variation in the CF microbial composition was patient-specific. The highly individualized nature of patient's lower airways microbiota is well-described in the CF literature (Whelan et al., 2017). We also identified that the stage of lung disease at the time of sample collection as a determinant of community structure when assessed as a single factor or concurrently with the PA strain type. This inverse correlation of microbial community diversity and lung function has been observed almost universally in CF microbiota studies (Coburn et al., 2015; Acosta et al., 2017). Indeed, work suggests that microbiota diversity serves as a better predictor for subsequent clinical outcomes than conventional culture-based microbial biomarkers (Acosta et al., 2018). We similarly observed variables such as sex, to be associated with the structure of the CF microbiota (Zhao et al., 2014), potentially important given the importance of sex as a determinant of CF outcomes (Nick et al., 2010). When alpha diversity was summarized using the SDI and samples were compared by the dominant taxa, we found that the dominance of the CF microbiome by classical CF pathogens such as *Pseudomonas* was associated with a decrease in alpha diversity. In contrast, samples dominated by *Streptococcus species* (i.e., *S. infantis*) showed the greatest diversity. This was in accordance with the observations of previous studies (Zhao et al., 2014; Coburn et al., 2015). These results are important because previous studies have shown that numerically dominant pathogens may influence the clinical outcomes (Sibley et al., 2010; Rogers et al., 2014).

In order to determine the microbial composition of the non-dominant members that may be obscured by the extreme dominance of *P. aeruginosa*, we sought to analyze the accessory microbiome. Here, we still found that samples clustered based on the PA strain type, although the percentage of the microbial community structure variation explained by that variable decreased (from 4.6 to 1.24%). Despite our inclusion of only patients who were positive by the Leeds criteria (Lee et al., 2003) for chronic *P. aeruginosa* infection, there were differences in the proportion of culture positive of *P. aeruginosa* at the time of sputum collection (Table 1). However, when we restricted our analysis to only those individuals who were *P. aeruginosa* positive in culture the same trends were observed with respect to the structure of the CF microbiota.

An important limitation of our study is that our findings relate to PES as a single example of ePA. Whether other described ePA strains may have the same associations with microbiome structure remains to be determined. Our use of V3-4 regions to identify microbiome constituents has only modest ability to identify taxonomic assignments to the species level. For that reason, it cannot be ruled out the possibility of infection of multiple *Streptococcus* species at the same time for those samples and that their dominant OTU was assigned to *Streptococcus* OTU, which would affect the observed alpha diversity differences. While our observed association of strain type and community structure is a novel observation, this does not confirm causation. While we were able to control for chronic infection status, we were not able to control for length of chronic *P. aeruginosa* infection in our cohort because samples/clinical data used in this study came exclusively from an adult clinic and our access to pediatric data limited. Accordingly, we were not able to assess whether the duration of *P. aeruginosa* infection may have an impact on the CF microbiome. Furthermore, how PES might shape the microbial community structure of the CF airway is unclear—if in fact it were responsible. As microbial interactions have been proposed to play a role in shaping *P. aeruginosa* evolution within the CF lower airways this could be through microbial secretions that may influence the fitness of other organisms either directly or indirectly, leading to potential positive or negative consequences (O'Brien and Fothergill, 2017). The use of animal models with transcriptomics and culture enhanced metagenomics may allow further granularity on this topic.

We observed that patients infected with PES, an ePA associated with worse CF clinical outcomes, have different bacterial community structures than those infected with uPA. While ePA may still be disproportionally more virulent than other PA strains as it has been previously reported, they may also function to modify the CF microbiome to one that is more harmful to the host by which patients infected with ePA experience worse lung clinical outcomes. Further study is required on the mechanism by which ePA might contribute to adverse outcomes.

## Data Availability Statement

The datasets generated for this study can be found in the National Center for Biotechnology Information (NCBI) Sequence Read Archive (SRA), BioProject ID PRJNA594982(http://www.ncbi.nlm.nih.gov/bioproject/594982).

## Ethics Statement

The studies involving human participants were reviewed and approved by University of Calgary Conjoint Health Research Ethics Board. The patients/participants provided their written informed consent to participate in this study.

## Author Contributions

NA was responsible for accessing sputum samples, extraction, wrote the initial draft of the manuscript, and microbiome related experiments. NA, BW, AH, RS, MS, and MW were responsible for microbiome analysis and statistical analyses. NA and MP were responsible for sample identification and clinical data collection. NA, BW, HR, and MP were responsible for maintenance of the CACFC biobank and clinical care records and documentation. MP, MS, and RS contributed conception and design of the study. All authors contributed to manuscript revision, read, and approved the submitted. MP supervised the project and is the guarantor of this work.

## Conflict of Interest

The authors declare that the research was conducted in the absence of any commercial or financial relationships that could be construed as a potential conflict of interest.
