# Supplementary material for: Cystic Fibrosis Patients Infected With Epidemic Pseudomonas aeruginosa Strains Have Unique Microbial Communities
Source: Front Cell Infect Microbiol. 2020 Apr 24;10:173. doi: 10.3389/fcimb.2020.00173 (PMC7212370; doi:10.3389/fcimb.2020.00173)
Supplement: Supplementary file 1 [file Table_1.docx]

Supplementary Material

# Supplementary Figures and Tables

## Supplementary Figures


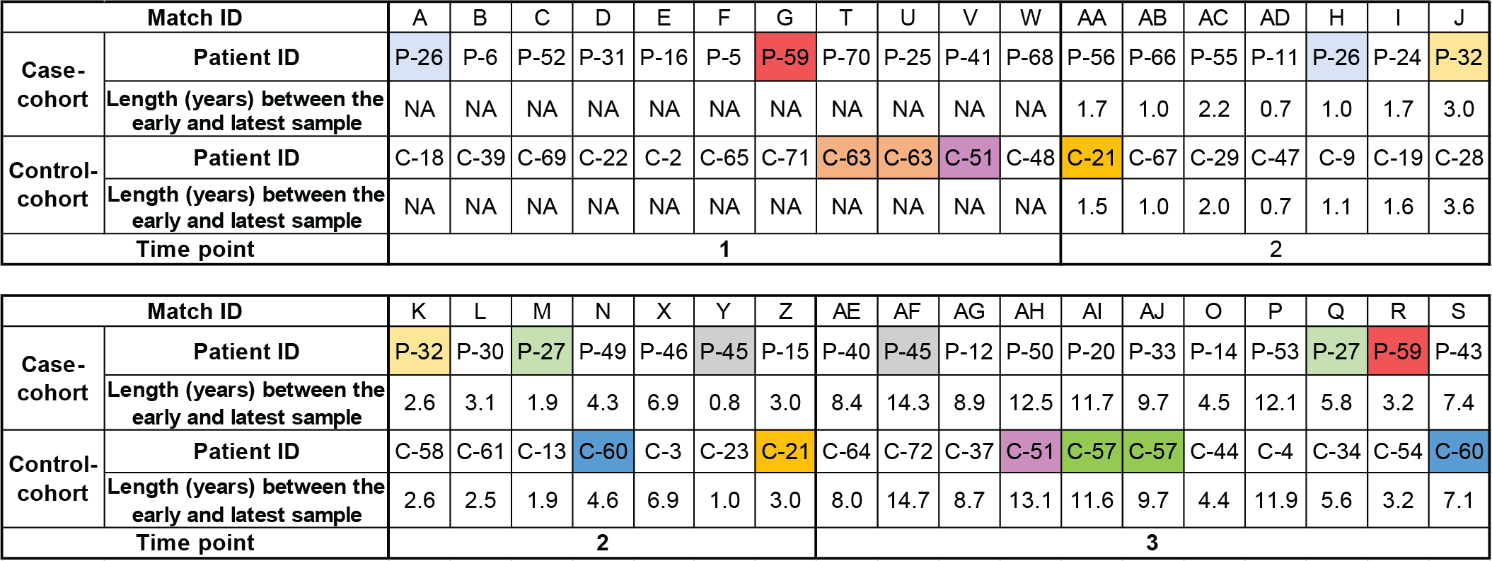


**Supplementary Figure 1.** **ePA-uPA match definition**. Thirty-six pairs of ePA-uPA match were identified in the study. Samples (i.e. 72 ePA and 72 uPA samples) were collected from up to three time points based on the availability of time of the patients (P-) or control (C-) samples including: 11 pairs that were assessed at only 1 time point, 14 pairs that were assessed at two time points and 11 pairs that were assessed for all three potential time points. The length (in years) between the early and latest sample used for those control-case pairs of samples that were study for more than one point was calculated. For patients that were assessed at only two time points the time span between these was a median 1.9 years (IQR: 1-3 years). For the patients that were analyzed at all three time points, samples were collected a median 8.8 years apart (IQR: 3.2-11.8 years). These match IDs correspond to the same that are stated on Fig 3. Case and cohort patients that were matched twice are color-coded. NA: not applicable.


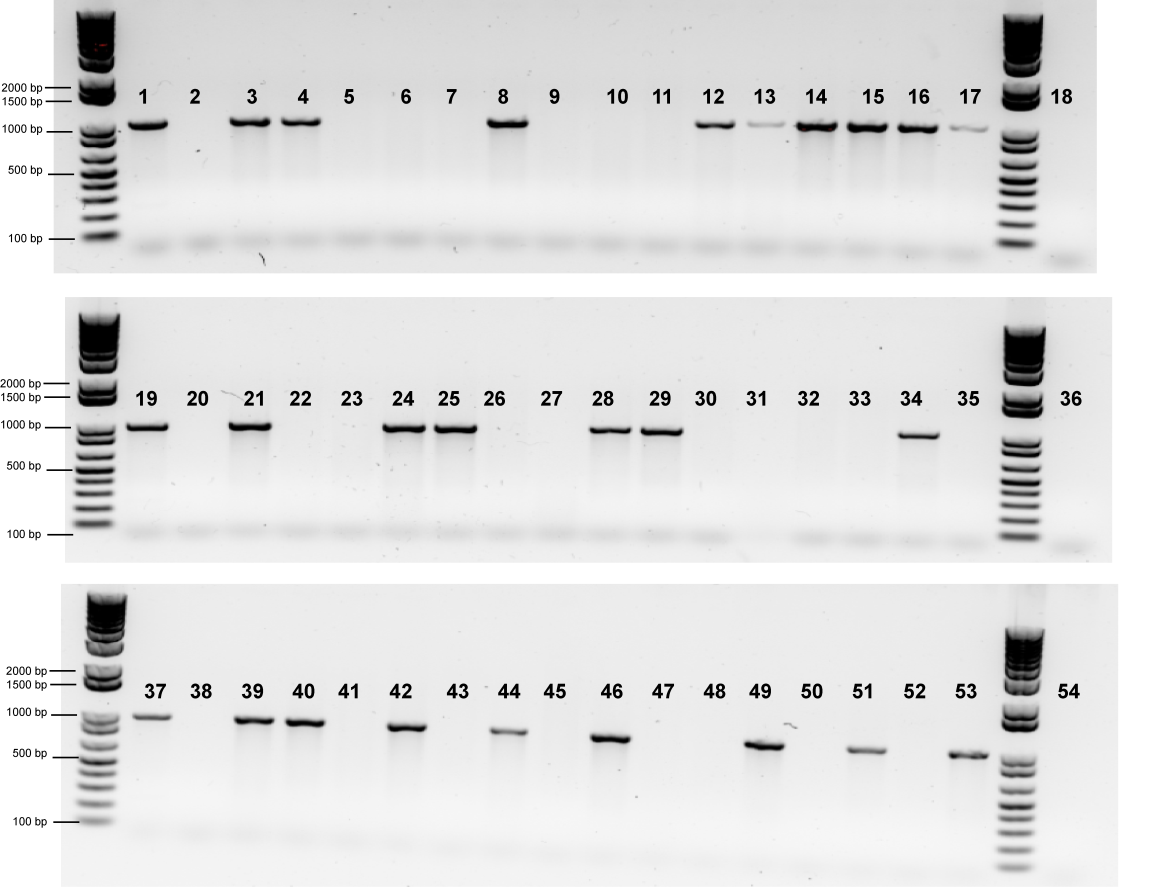


**Supplementary Figure 2.** **PCR PES confirmation.** Products from samples from some patients included in this study infected with PES ePA (lanes 3, 4, 8, 12, 13, 14, 15, 16, 17, 21, 24, 25, 28, 29, 34, 39, 40, 42, 44, 46, 49, 51 and 53) and uPA (lanes 5, 6, 7, 9, 10, 11, 22, 23, 26, 27, 30, 31, 32, 33, 35, 41, 43, 45, 47, 48, 50 and 52). Positive control: PES isolate (lanes 1, 19 and 37), negative control: *P. aeruginosa* strain PAO1 (lanes 2, 20 and 38) and blank for PCR: H_2_O (lanes 18, 36 and 54). ePA: epidemic strains of *P. aeruginosa* and uPA: unique strains of *P. aeruginosa.*

**
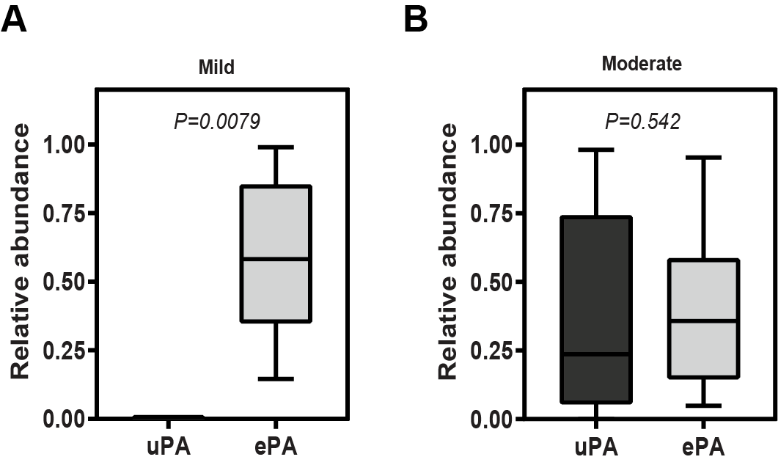
**

**Supplementary Figure 3.** **Relative abundance of *Pseudomonas* OTU reads between ePA and uPA samples.** Wilcoxon rank-sum (Mann-Whitney) tests were performed in the mild (**A**) and in the moderate (**B**) group based on the stage of lung disease. The median and interquartile ranges (IQR) are indicated as the middle, top and bottom lines of each box. Ends of the whiskers mark the min and max. ePA: epidemic strains of *P. aeruginosa*, uPA: unique strains of *P. aeruginosa,* and OTU: operational taxonomic unit.

## Supplementary Tables

**Supplementary Table 1.** CF patients that were matched twice in the study.

| **Cohort** | **Patient ID** | **Match ID**^†^ | **Time point** | **Date collected** |
| --- | --- | --- | --- | --- |
| **Control-cohort** | **C-21** | Z | T1 | 24/02/2004 |
|  |  | Z | T2 | 21/02/2007 |
|  |  | AA* | T1 | 17/12/2007 |
|  |  | AA* | T2 | 15/06/2009 |
|  | **C-51** | V | T1 | 21/07/1999 |
|  |  | AH* | T1 | 21/06/1999 |
|  |  | AH* | T2 | 06/11/2006 |
|  |  | AH* | T3 | 23/07/2012 |
|  | **C-57** | AI | T1 | 11/09/2000 |
|  |  | AI | T2 | 07/06/2006 |
|  |  | AI | T3 | 25/04/2012 |
|  |  | AJ* | T1 | 28/03/2001 |
|  |  | AJ* | T2 | 06/11/2006 |
|  |  | AJ* | T3 | 07/12/2010 |
|  | **C-60** | N | T1 | 04/04/1999 |
|  |  | N | T2 | 20/11/2003 |
|  |  | S* | T1 | 06/07/2007 |
|  |  | S* | T2 | 13/10/2010 |
|  |  | S* | T3 | 28/07/2014 |
|  | **C-63** | T | T1 | 04/11/1998 |
|  |  | U* | T1 | 22/03/2000 |
| **Case-cohort** | **P-26** | A | T1 | 14/04/2004 |
|  |  | H* | T1 | 09/04/2014 |
|  |  | H* | T2 | 15/04/2015 |
|  | **P-27** | M | T1 | 06/06/2007 |
|  |  | M | T2 | 04/05/2009 |
|  |  | Q* | T1 | 14/01/2008 |
|  |  | Q* | T2 | 08/09/2010 |
|  |  | Q* | T3 | 23/10/2013 |
|  | **P-32** | J | T1 | 07/10/2008 |
|  |  | J | T2 | 24/10/2011 |
|  |  | K* | T1 | 15/09/2009 |
|  |  | K* | T2 | 07/05/2012 |
|  | **P-45** | AF | T1 | 07/03/2000 |
|  |  | AF | T2 | 27/06/2007 |
|  |  | AF | T3 | 23/06/2014 |
|  |  | Y* | T1 | 14/05/2012 |
|  |  | Y* | T2 | 20/03/2013 |
|  | **P-59** | R | T1 | 08/12/1998 |
|  |  | R | T2 | 19/01/2000 |
|  |  | R | T3 | 06/03/2002 |
|  |  | G* | T1 | 13/02/2002 |

*These matches were excluded for the sensitivity analysis done in order to have only one match per patient or control.

^†^ These match IDs correspond to the same that are stated on Fig 3.

**Supplementary Table 2.** CF bacterial community structure variation explained by various factors and their interactions based on Bray-Curtis distances when samples were rarefied to a sequence depth of 15,000 sequences per sample.

| Variable | | F | R^2^ (%) | P-value^*^ |
| --- | --- | --- | --- | --- |
| Single Factor | A) PA strain type | 21.3 | 4.8 | 0.002 |
|  | B) FEV_1_% | 7.25 | 1.6 | 0.002 |
|  | C) Sex | 12.8 | 2.9 | 0.002 |
|  | D) Stage of lung disease^†^ | 2.87 | 3.9 | 0.014 |
|  | E) Patient | 4.1 | 55.8 | 0.002 |
| Interaction | A x B | 1.89 | 1.2 | 0.085 |
|  | A x D | 2.33 | 3.06 | 0.017 |

*P-values determined by PERMANOVA and they were adjusted for multiple testing by using the procedure of Benjamini and Hochberg (false discovery rate threshold, 5%).

An “x” represents interaction between variables that are letter-coded.

^†^Stages of lung disease: mild (>80%), moderate (40-80%), and advanced (<40 %).

PA: *P. aeruginosa*

**Supplementary Table 3.** CF bacterial community structure variation explained by various factors and their interactions based on Bray-Curtis distances when samples were rarefied to a sequence depth of 15,000 sequences per sample, using the accessory microbiome data set.

| Variable | | F | R^2^ (%) | P-value^*^ |
| --- | --- | --- | --- | --- |
| Single Factor | A) PA strain type | 4.25 | 1.68 | 0.003 |
|  | B) FEV_1_% | 1.9 | 0.76 | 0.113 |
|  | C) Sex | 2.34 | 0.92 | 0.074 |
|  | D) Stage of lung disease^†^ | 1.3 | 2.4 | 0.223 |
|  | E) Patient | 3.41 | 70.4 | 0.003 |
| Interaction | A x B | 2.17 | 1.96 | 0.074 |
|  | A x D | 1.43 | 2.6 | 0.150 |

*P-values determined by PERMANOVA and they were adjusted for multiple testing by using the procedure of Benjamini and Hochberg (false discovery rate threshold, 5%).

An “x” represents interaction between variables that are letter-coded.

^†^Stages of lung disease: mild (>80%), moderate (40-80%), and advanced (<40 %).

PA: *P. aeruginosa*

**Supplementary Table 4.** CF bacterial community structure variation explained by various factors and their interactions based on Bray-Curtis distances when samples were rarefied to a sequence depth of 15,000 sequences per sample, in only samples that were PA positive in culture.

| Variable | | F | R^2^ (%) | P-value^*^ |
| --- | --- | --- | --- | --- |
| Single Factor | A) PA strain type | 9.03 | 2.45 | 0.002 |
|  | B) FEV_1_% | 9.21 | 2.5 | 0.002 |
|  | C) Sex | 8.96 | 2.43 | 0.002 |
|  | D) Stage of lung disease^†^ | 2.43 | 4.21 | 0.026 |
|  | E) Patient | 4.37 | 63.01 | 0.002 |
|  | F) Mucoid PA | 2.38 | 2.08 | 0.057 |
| Interaction | A x B | 1.62 | 1.37 | 0.142 |
|  | A x D | 2.11 | 3.53 | 0.028 |

*P-values determined by PERMANOVA and they were adjusted for multiple testing by using the procedure of Benjamini and Hochberg (false discovery rate threshold, 5%).

An “x” represents interaction between variables that are letter-coded.

^†^Stages of lung disease: mild (>80%), moderate (40-80%), and advanced (<40 %).

PA: *P. aeruginosa*
